# Why Is School Closed Today? Unplanned K-12 School Closures in the United States, 2011–2013

**DOI:** 10.1371/journal.pone.0113755

**Published:** 2014-12-02

**Authors:** Karen K. Wong, Jianrong Shi, Hongjiang Gao, Yenlik A. Zheteyeva, Kimberly Lane, Daphne Copeland, Jennifer Hendricks, LaFrancis McMurray, Kellye Sliger, Jeanette J. Rainey, Amra Uzicanin

**Affiliations:** 1 Division of Global Migration and Quarantine, Centers for Disease Control and Prevention, Atlanta, Georgia, United States of America; 2 Chenega Government Consulting, Chesapeake, Virginia, United States of America; 3 Oak Ridge Associated Universities, Oak Ridge, Tennessee, United States of America; The Australian National University, Australia

## Abstract

**Introduction:**

We describe characteristics of unplanned school closures (USCs) in the United States over two consecutive academic years during a non-pandemic period to provide context for implementation of school closures during a pandemic.

**Methods:**

From August 1, 2011 through June 30, 2013, daily systematic internet searches were conducted for publicly announced USCs lasting ≥1 day. The reason for closure and the closure dates were recorded. Information on school characteristics was obtained from the National Center for Education Statistics.

**Results:**

During the two-year study period, 20,723 USCs were identified affecting 27,066,426 students. Common causes of closure included weather (79%), natural disasters (14%), and problems with school buildings or utilities (4%). Only 771 (4%) USCs lasted ≥4 school days. Illness was the cause of 212 (1%) USCs; of these, 126 (59%) were related to respiratory illnesses and showed seasonal variation with peaks in February 2012 and January 2013.

**Conclusions:**

USCs are common events resulting in missed school days for millions of students. Illness causes few USCs compared with weather and natural disasters. Few communities have experience with prolonged closures for illness.

## Introduction

Children play a significant role in transmitting influenza virus within the socially dense school environment and in introducing influenza into their households [1], [2]. Thus, closing schools before influenza transmission becomes widespread in schools and surrounding communities may be recommended as a community strategy to slow progression of a severe influenza pandemic [3], [4]. In addition to these preemptive closures, school closures may be implemented reactively due to high levels of student and staff absenteeism related with widespread influenza transmission, as previously reported during local epidemics of seasonal influenza [5], [6] and during influenza pandemics [7], [8]. During the second wave of the influenza A(H1N1)pdm09 pandemic in the United States, which coincided with the start of the 2009–2010 school year, 812 school closure events were reported to a national surveillance system developed to monitor pandemic-related closure events from direct reports, state monitoring systems, and media scans and online searches [9]. This surveillance system provided important situational awareness with regard to pandemic-related school dismissals, including the geographic distribution, the number of school days missed, and the number of students affected by dismissal events.

Characteristics of unplanned school closures due to causes other than 2009 influenza A(H1N1)pdm09 pandemic have not been previously described for the United States. Monitoring and analyzing the patterns and characteristics of all unplanned school closures (USCs) in the United States that occur outside of an influenza pandemic can help describe the baseline experience of communities with school closures. This provides context for how communities may perceive and cope with the preemptive school closures that may be warranted as a community mitigation strategy during an influenza pandemic to slow disease transmission. In this study, we identify publicly announced USCs lasting ≥1 school day in the United States during two consecutive non-pandemic school years (2011–2012 and 2012–2013) and describe the characteristics of these closures.

## Methods

### Search strategy

From August 1, 2011 through June 30, 2013, daily systematic searches of Google, Google News, and Lexis-Nexis were conducted to identify potential unplanned school closure events. Searches in Google and Google News were conducted for the previous 24 hours in the United States using the following terms: “school closed,” “schools closed,” “schools are closed,” and “schools will be closed.” Lexis-Nexis searches were conducted daily for full text articles in English according to the following search query: *headline(academy or school) and headline(close or closed or closing or closings or closure or closures or closes or dismiss!) and date aft July 31, 2011*. Whether a school closure was planned or unplanned was verified against the official website for the school or school district.

USCs were defined as the decision by the school district or by an individual school to close for ≥1 school day. Both public and private school closure events were included in the study. Closures associated with scheduled school holidays (e.g., Thanksgiving Day holiday, winter break) were considered planned closures and therefore not included in this analysis. For closures that spanned both unplanned and planned closure days, such as those contiguous with weekends or planned holidays, only unplanned closure days were included in the analysis.

### Data on unplanned school closure events

We used the information provided in school closure announcements to abstract relevant data, including the scope of closure (individual school versus district-wide), number of school days missed, and reason for closure, into a Microsoft Access database. District-wide closures were those that occurred at the level of the school district, where all or most of the schools within the district closed. In contrast, individual-level closure events were decisions made by individual schools to close, without closing other schools in the district. We counted the number of school days missed based on the announced dates of school closing and reopening (i.e., weekends were excluded). For school closure announcements that did not specify the reopening date or the duration of closure, we assumed that the closure had lasted for only one school day. We also performed a sensitivity analysis limited to the subset of closure events for which the reopening date was specified.

Reasons for closure were classified according to the following categories: weather, natural disaster, school building or utilities problem, violence, illness, environmental problem, teacher strike, death of staff or student, and other reasons. In this analysis, natural disasters included earthquakes, large floods, hurricanes, tornados, and wildfires; weather events included fog, ice or snow storms, rain storms, wind, extreme temperatures, and other weather events. Structural problems or utility failures affecting the school building were classified as problems with the school building or utilities. Violence-related closures included threats against students or staff, bomb threats, a shooting at the school, a shooting in the surrounding area, and other violent events. Closures were classified as due to environmental problems for non-structural problems with the school environment posing a potential health risk, such as asbestos, fumes, or an animal infestation. Illness-related closures were due to known causes of illness among students or staff, such as influenza, norovirus, or methicillin-resistant *Staphylococcus aureus*, or unknown illnesses described by symptoms, such as dizziness.

### School and district data

We obtained publicly available information on the characteristics of private and public schools and public school districts, including number of students and staff, rural or non-rural (city, suburb, or town) locale, and number of students eligible for free or reduced lunch (for public schools only), for the 2010–2011 or 2011–2012 school year from the National Center for Education Statistics (NCES) [10]. The most recent year of data available was used for each school. Mapping files for school districts were also obtained from NCES. Each district-wide or individual-level school closure in the Google and Lexis-Nexis search results was matched to an NCES district or school identification number based on the district or school name, city, and state.

We categorized schools according to the grades enrolled at that school. Elementary school grades were defined as kindergarten through 5^th^ grade (approximate ages 5–10 years); middle school grades were 6–8^th^ (approximate ages 11–13 years), and high school grades were 9–12^th^ (approximate ages 14–18 years). Schools serving grades spanning more than one category were categorized as elementary–middle, elementary–high, or middle–high. As this analysis focused on grades K–12, individual schools that included only pre-kindergarten students were excluded from the analysis of all USCs.

### Analysis

We described characteristics of USCs according to the data abstracted from public announcements. To describe school characteristics affected by USCs, we matched USCs to corresponding NCES school and district data where possible using a unique school or district identification number. Analysis was conducted using SAS 9.3 (Cary, NC). For bivariate analyses we used PROC SURVEYLOGISTIC, a SAS procedure that accounts for clustering of results by specific events (e.g., Hurricane Sandy). We used the Rao-Scott chi-square test to evaluate differences between two or more proportions. All p-values are two-sided, and P<.05 was considered statistically significant. Maps were created using R 3.0.1 [11], with packages sp [12], [13] and rgdal [14].

### Ethics statement

The project underwent ethical review at the Centers for Disease Control and Prevention and was determined not to involve human subjects; it was therefore not subject to institutional review board review requirements.

## Results

There were 20,723 closure events recorded from August 1, 2011 through June 30, 2013; of these, 4,390 occurred during 2011–2012, and 16,333 occurred during 2012–2013 (Table 1). The median number of school closure days was 1 day (range [IQR]: 1–20). Closure events were matched to 58,330 schools, of which 52,918 (91%) were public schools, 4,607 (8%) were private schools, and 805 (1%) were USCs that did not match to schools in the NCES database but were assumed to affect at least one school. As some schools experienced multiple closure events, there were 49,898 unique schools over two academic years that matched to schools in the NCES database, representing 39% of the schools in the NCES database. Most of the schools affected were classified as elementary (20,523; 35%) or elementary-middle schools (13,342; 23%). Overall, 18,116 (31%) schools were located in rural areas, and 39,242 (67%) were located in cities, suburbs, or towns. The median number of students affected per district-wide closure was 476, and the median number of students affected per individual-level school closure was 259. Forty-eight percent of public school students affected by USCs were eligible for free or reduced price lunch at school. In comparison, there were 126,573 schools in the NCES database, of which 98,816 (78%) were public schools and 27,757 (22%) were private schools. The most common school classifications in the NCES database were elementary (42,624; 33%) and elementary-middle schools (32,345; 26%), and 37,091 (29%) of schools were located in rural areas.

**Table 1:** Characteristics of school closures by academic year — United States, 2011–2013.

	2011–2012	2012–2013	Total
Number of closure events	n = 4,390	n = 16,333	N = 20,723
Type of closure, n (%)			
District-wide	2,846 (65)	10,985 (67)	13,831 (67)
Individual-level	1,544 (35)	5,348 (33)	6,892 (33)
Season, n (%)			
Fall (Sep–Nov)	773 (18)	2,733 (17)	3,506 (17)
Winter (Dec–Feb)	2,307 (53)	8,775 (54)	11,082 (53)
Spring (Mar–May)	824 (19)	4,810 (29)	5,634 (27)
Summer (Jun–Aug)	486 (11)	15 (<1)	501 (2)
Cause, n (%)			
Weather	3,235 (74)	13,234 (81)	16,469 (79)
Natural disaster	530 (12)	2,315 (14)	2,845 (14)
School building/utilities problem	413 (9)	432 (3)	845 (4)
Violence	38 (1)	111 (1)	149 (1)
Illness	69 (2)	143 (1)	212 (1)
Respiratory illness	34 (1)	92 (1)	126 (1)
GI illness	23 (1)	20 (<1)	43 (<1)
Unknown illness	8 (<1)	27 (<1)	35 (<1)
Meningitis illness	1 (<1)	2 (<1)	3 (<1)
Other illness	3 (<1)	2 (<1)	5 (<1)
Environmental problem	52 (1)	17 (<1)	69 (<1)
Teacher strike	16 (<1)	9 (<1)	25 (<1)
Death of staff or student	13 (<1)	12 (<1)	25 (<1)
Other	24 (1)	60 (<1)	84 (<1)
Unplanned closure days, median (range)^a^	1 (1–20)	1 (1–12)	1 (1–20)
≥4 days	173 (4)	598 (4)	771 (4)
No. of students affected^b^	5,687,554	21,378,872	27,066,426
No. of teachers affected^b^^,^ c	342,139	1,394,197	1,736,337
Number of schools affected^d^	n = 11,717	n = 46,613	N = 58,330
Public schools	10,675 (91)	42,243 (91)	52,918 (91)
Private schools	778 (7)	3,829 (8)	4,607 (8)
Unknown	264 (2)	541 (1)	805 (1)
Type of school, n (%)			
Elementary school (K–5 gr)	4,143 (35)	16,380 (35)	20,523 (35)
Elementary–middle school (K–8 gr)	2,483 (21)	10,859 (23)	13,342 (23)
Elementary–high school (K–12 gr)	797 (7)	2,304 (5)	3,101 (5)
Middle school (6–8 gr)	1,312 (11)	5,408 (12)	6,720 (12)
Middle–high school (6–12 gr)	858 (7)	2,718 (6)	3,576 (6)
High school (9–12 gr)	1,787 (15)	7,934 (17)	9,721 (17)
Type of school not specified	337 (3)	1,010 (2)	1,347 (2)
School setting, n (%)			
City	2,840 (24)	11,402 (24)	14,242 (24)
Suburb	3,194 (27)	14,435 (31)	17,629 (30)
Town	1,573 (13)	5,798 (12)	7,371 (13)
Rural	3,891 (33)	14,225 (31)	18,116 (31)
Not specified	219 (2)	753 (2)	972 (2)
Percent of students eligible for free/reduced lunch, median (IQR)^e^	56 (35–76)	45 (24–68)	48 (26–70)

a Closure events where reopening date not specified were assumed to last 1 day.

b Students and teachers were counted once for each closure event.

c Part-time teaching positions were reported as a fraction of one full-time position [30].

d Schools were counted once for each closure event. 286 districts that did not match to NCES schools were counted as one school. District-wide closures could only match to public schools in NCES database.

e Reported for 10,207 schools in 2011–2012 and 40,620 schools in 2012–2012; data applicable for public schools only.

The most frequent causes of closure events overall were weather (16,469; 79%) and natural disasters (2,845; 14%). Problems with the school building or utilities caused 10% of individual-level school closures and 1% of district-wide closures. Violence was cited as the reason for 1% of all school closure events. There were 212 illness-related school closure events comprising 1% of all school closure events; 126 (59%) of illness-related closures were due to respiratory illness, including influenza.

**Figure 1:**
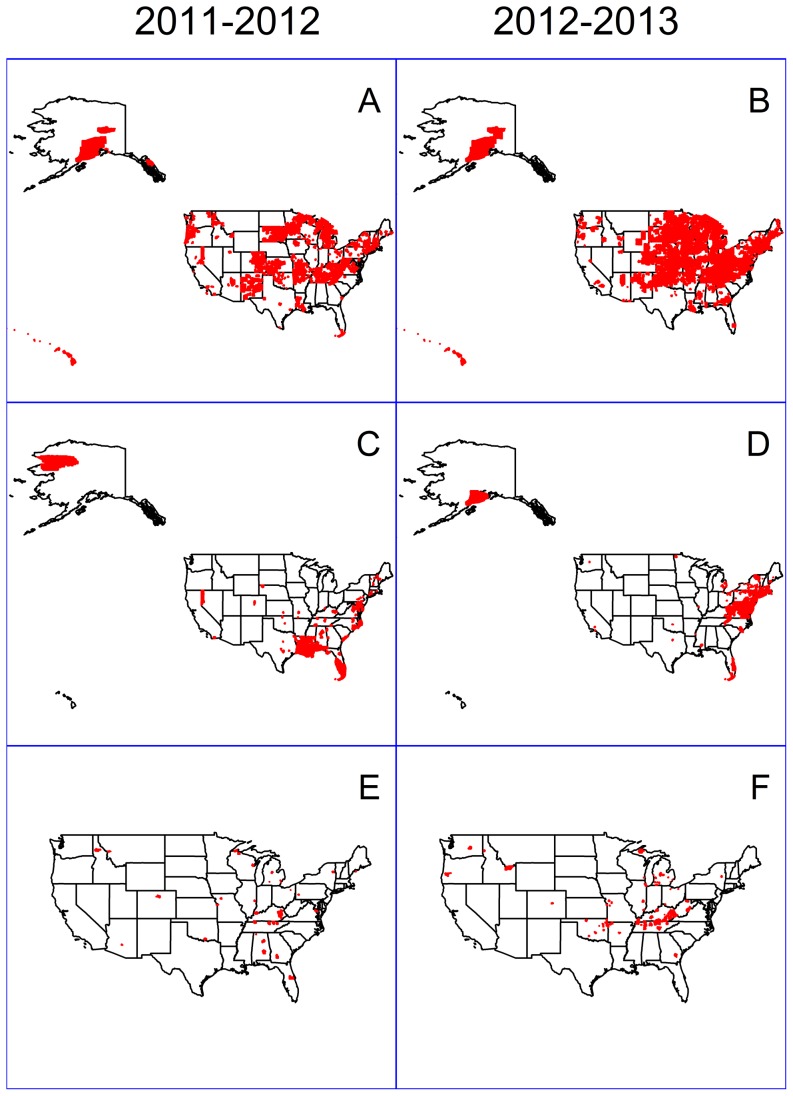
Geographic distribution of public school districts with closure events related to weather, natural disasters, and illness, by year — United States, August 2011–June 2013. Panels A and B: weather-related closure events; panels C and D: natural disaster-related closure events; panels E and F: illness-related closure events, one event in Alaska not shown.

School closures occurred throughout the United States (Figure 1). Weather-related closures occurred over a wide geographic distribution, with some sparing of the mountain region states, California, and Texas. Closures related to natural disasters were clustered in Louisiana and Florida during 2011–2012 and in the northeastern states during 2012–2013. With the exception of some clustering of illness-related closures in Kentucky and Tennessee during 2012–2013, there was no clear pattern to the geographic distribution of illness-related closure events.

School closure events were more frequently district-wide when the cause was weather (11,415/16,469; 69%), natural disaster (1,988/2,845; 70%), or illness (120/212; 57%); closure events occurred more often at the individual school level when the cause was due to problems with the building or utilities (693/845; 82%) or violence (90/149; 60%) (Table 2). Weather-related closures occurred most often in winter (10,516/16,469; 64%), whereas the majority of natural disaster-related closures (2,363/2,845; 83%) occurred during the fall. Winter was also the most common season for illness-related (143/212; 67%) and violence-related closures (89/149; 60%). Closures related to natural disasters had the highest percentage of events causing ≥4 unplanned closure days (550/2,845; 19%) compared with closures related to weather (176/16,469; 1%), building or utility problems (18/845; 2%), illness (7/212; 3%), and violence (2/149; 1%). Most illness-related closures involved 1 (153/212; 72%) or 2 (43/212; 20%) unplanned closure days. Among 63 respiratory illness-related closures where the date of reopening was known, 59 (94%) resulted in <4 missed school days.

**Table 2:** Selected characteristics of school closure events and schools by reason for school closure — United States, 2011–2013.

	Weather	Natural disasters	Building/utilities	Illness	Violence
	n = 16469	n = 2845	n = 845	n = 212	n = 149
School year, n (%)					
2011–2012	3235 (20)	530 (19)	413 (49)	69 (33)	38 (26)
2012–2013	13234 (80)	2315 (81)	432 (51)	143 (67)	111 (74)
Type of closure, n (%)					
District-wide	11415 (69)	1988 (70)	152 (18)	120 (57)	59 (40)
Individual-level	5054 (31)	857 (30)	693 (82)	92 (43)	90 (60)
Season, n (%)					
Fall (Sep–Nov)	715 (4)	2363 (83)	316 (37)	23 (11)	19 (13)
Winter (Dec–Feb)	10516 (64)	24 (1)	264 (31)	143 (67)	89 (60)
Spring (Mar–May)	5233 (32)	31 (1)	209 (25)	45 (21)	39 (26)
Summer (Jun–Aug)	5 (<1)	427 (15)	56 (7)	1 (<1)	2 (1)
School days lost^a^, n (%)					
<4 days	16293 (99)	2295 (81)	827 (98)	205 (97)	147 (99)
1 day	15343 (93)	1790 (63)	765 (91)	153 (72)	132 (89)
2 days	774 (5)	391 (14)	46 (5)	43 (20)	10 (7)
3 days	176 (1)	114 (4)	16 (2)	9 (4)	5 (3)
≥4 days	176 (1)	550 (19)	18 (2)	7 (3)	2 (1)
Students affected per event^b^					
<500	9019 (62)	1196 (46)	413 (51)	134 (69)	68 (47)
≥500	5606 (38)	1424 (54)	389 (49)	60 (31)	76 (53)
Number of schools^c^	n = 42291	n = 12810	n = 1386	n = 443	n = 304
School setting, n (%)					
City, suburb, or town	26340 (62)	10492 (82)	967 (70)	279 (63)	203 (67)
Rural	15216 (36)	2141 (17)	388 (28)	153 (35)	100 (33)
Unknown	735 (2)	177 (1)	31 (2)	11 (2)	1 (<1)
Type of school, n (%)					
Elementary school	14641 (35)	4948 (39)	479 (35)	147 (33)	97 (32)
Elementary–middle school	9916 (23)	2448 (19)	299 (22)	102 (23)	52 (17)
Elementary–high school	2377 (6)	584 (5)	69 (5)	26 (6)	18 (6)
Middle school	4817 (11)	1580 (12)	166 (12)	52 (12)	33 (11)
Middle–high school	2607 (6)	780 (6)	88 (6)	31 (7)	23 (8)
High school	7053 (17)	2065 (16)	254 (18)	74 (17)	80 (26)
Type of school not specified	880 (2)	405 (3)	31 (2)	11 (2)	1 (<1)

a Closure events where reopening date not specified were assumed to last 1 day.

b Percents reported out of number of events where data on number of students affected were known: 194 illness-, 2620 natural disaster-, 802 building/utilities-, 144 violence-, and 14625 weather-related events.

c Schools were counted once for each closure event.

Compared with weather-related closures, closures related to natural disasters (OR: 22.2, 95% CI: 7.7–64.1), environmental problems (OR: 12.1, 95% CI: 3.1–47.5), and teacher strikes (OR: 43.6, 95% CI: 11.6–163.3) were more likely to result in ≥4 unplanned closure days (Table 3). District-wide closures were also more likely than individual-level closures to be associated with ≥4 unplanned closure days. Illness-related school closures were more likely to begin on Thursdays or Fridays when compared to weather-related closures (P<.001).

**Table 3:** Selected characteristics of school closure events and association with ≥4 unplanned closure days — United States, 2011–2013.

	≥4 unplanned closure days, n/N (%)	OR (95% CI)*
**Cause**		
Weather	176/16,469 (1)	ref
Natural disasters	550/2,845 (19)	22.2 (7.7–64.1)
School building/utilities problem	18/845 (2)	2.0 (0.7–5.7)
Violence	2/149 (1)	1.3 (0.4–3.6)
Illness	7/212 (3)	3.2 (0.9–10.7)
Environmental problem	8/69 (12)	12.1 (3.1–47.5)
Teacher strike	8/25 (32)	43.6 (11.6–163.3)
Death of staff/student	1/25 (4)	3.9 (0.4–40.1)
Other	1/84 (1)	1.1 (0.1–10.2)
**Type of closure**		
Individual	592/13,831 (4)	ref
District	179/6,892 (3)	1.7 (1.4–2.0)

*Accounts for clustering by specific events.

We performed a sensitivity analysis limited to the subset of closures for which the reopening date was specified. Of the 18,344 closures that were assumed to be one-day events, 6363 (35%) were confirmed as one-day closures, and 11,981 (65%) were assumed to be one-day events because the reopening date was not specified. Of the 8742 closure events for which re-opening date was known, closures related to natural disasters (OR: 27.1, 95% CI: 9.0–81.4), environmental problems (OR: 9.5, 95% CI: 2.0–43.9), and teacher strikes (OR: 18.3, 95% CI: 4.8–70.8) remained more likely to result in ≥4 unplanned closure days compared with weather-related events. District-wide closures remained more likely than individual-level closures to be associated with ≥4 unplanned closure days.

Several prominent events, including Hurricanes Irene, Isaac, and Sandy, contributed substantially to the number of student-days lost. The largest natural disaster event was Hurricane Sandy in October 2012: twenty states reported 1647 district-wide and 637 individual-level closures resulting in 13,759,663 student-days lost (Table 4). Hurricane Isaac in August 2012 was associated with closures reported from four states and resulted in 3,875,386 lost student-days. In contrast, Hurricane Irene in August 2011 was associated with closures in 14 states, but resulted in fewer lost student-days (260,981). Outside of weather- and natural disaster-related events, the Chicago teacher strike in September 2012 resulted in the most student-days lost, causing 8 district-wide closures and 3,279,110 lost student-days.

**Table 4:** Selected events causing school closures — United States, 2011–2013.

Event	Consequences^a^
Hurricane Irene (Aug 2011)	14 states reported closures
	117 district-wide and 19 individual-level closures
	260,981 student-days lost
Respiratory illness, 2011–2012	11 states reported
	closures18 district-wide and 16 individual-level closures
	14,357 student-days lost
Hurricane Isaac (Aug 2012)	4 states reported closures
	148 district-wide and 115 individual-level closures
	3,875,386 student-days lost
Chicago teacher strikes (Sep 2012)	8 district-wide closures
	3,279,110 student-days lost
Hurricane Sandy (Oct 2012)	20 states reported closures
	1647 district-wide and 637 individual-level closures
	13,758,663 student-days lost
Respiratory illness, 2012–2013	18 states reported closures
	62 district-wide and 30 individual-level closures
	59,366 student-days lost

a Student-days lost calculated by multiplying number of students per closure event [10] by number of unplanned closure days. Students were counted once for each closure event. Closure events where reopening date not specified were assumed to last 1 day.

**Figure 2:**
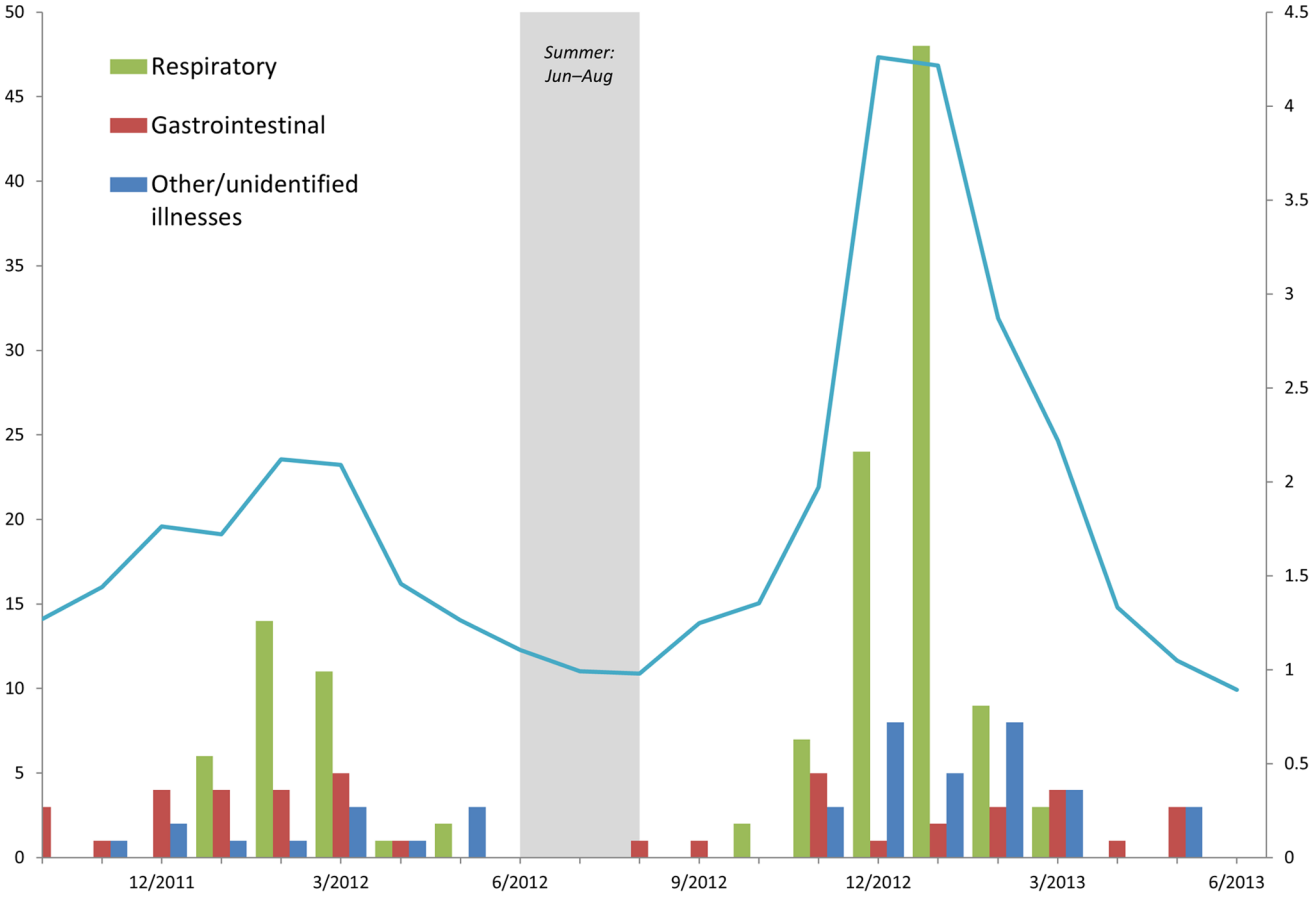
Number of illness-related school closure events by week and type of illness and national percent of medical provider visits for influenza-like illness — United States, 2011–2013. *National percent of medical provider visits for influenza-like illness [15].

The number of illness-related school closure events varied by type of illness syndrome and time of year (Figure 2). For respiratory illness-related closures, there was a peak in the number of closure events during the winter (February 2012 and January 2013). School closures related to gastrointestinal illness did not show a clear seasonal pattern. More respiratory illness-related closures occurred during the 2012–2013 school year compared with the 2011–2012 school year; this followed the pattern of percent of medical provider visits for influenza-like illness nationally [15]. Overall, respiratory illness, including influenza, resulted in 14,357 student-days lost during 2011–2012 and 59,366 student-days lost in 2012–2013.

## Discussion

Unplanned school closures affect students, communities, and families every year, and understanding the characteristics of these non-pandemic closures, including their location, frequency, and duration, can help in anticipating how communities may cope with preemptive or reactive school closures during a pandemic. Over the two-year study period, more than 27 million students and 1.7 million teachers over a wide geographic area were affected by school closure events lasting ≥1 day. Most frequently, these closures were related to weather events, such as ice and snow storms, or natural disasters, such as hurricanes and wildfires. Most USCs result in few missed school days, but closures resulting in several missed school days were also observed, such as those associated with Hurricane Sandy in 2012. Illness-related closures were rare; of these, over half were found to be due to respiratory illness.

This study presents a view of several notable events that caused USCs affecting a large number of students during 2011–2013. These events included highly visible natural disasters such as Hurricanes Isaac and Sandy, as well major community events such as the Chicago teachers' strike. The winter of 2012–2013 was marked by more adverse weather events compared with the relatively mild winter of 2011–2012 [16], and the pattern of weather-related school closures reflects this difference. Hurricanes Irene (August 2011), Isaac (August 2012), and Sandy (October 2012) all caused major disruptions in the community [17]–[20], and all resulted in USCs in multiple affected states. Weather and natural disasters, which underlie the majority of USCs, can cause wide variation in the number of USCs from year to year. By tracking public reports of school closure events, we were able to detect, quantify, and describe certain effects of these notable events on school-aged children.

Although less common than closures related to weather or natural disasters, illness-related USCs were also reported, and over half of these illness-related USCs were due to respiratory illness. The pattern of respiratory illness-related USCs reflected national influenza activity. The 2011–2012 season was a relatively mild influenza season, during which the percent of medical visits for influenza-like illness (ILI) peaked at only 2.4% [15]. In contrast, the 2012–2013 influenza season was a more typical influenza season, with the percent of medical visits for ILI peaking at 6.1%. The pattern of school closures for respiratory illness reflected this trend, with fewer respiratory illness-related USCs reported in 2011–2012 compared with 2012–2013. This approach of monitoring respiratory illness-related USCs from publicly available data may be a useful complement to existing disease surveillance systems as it reflects national trends and additionally provides real-time, hyperlocal reports of school closures associated with respiratory disease activity among school-aged children, which may be useful during outbreaks of respiratory infectious diseases, including pandemic and seasonal influenza, to describe effects on schools and communities.

During the 2009 pandemic, surveillance for pandemic-related school closures found that 81% of USCs resulted in <4 missed school days [9]. This differs from the pattern seen with respiratory illness-related closures in this study, which found that 94% of illness-related closures resulted in <4 missed school days. The respiratory illness-related USCs in this study may include outbreaks of non-influenza respiratory illness; however, another explanation for this finding is that differences in the level of community concern during the pandemic compared with a non-pandemic period may have contributed to the decision on the number of days to close schools.

Despite these differences, examining the characteristics of USCs and the student-days lost may offer useful insights for planning for preemptive school closures as a pandemic mitigation strategy. This study found that short USCs occur frequently, suggesting that communities may be accustomed to coping with school closures of short duration. In contrast, many simulation studies of school closure policies suggest that during the early phase of an evolving pandemic, closures lasting two weeks or more may be necessary to delay the local spread of the pandemic [21]. During the 2009 H1N1 pandemic, few parents reported that short, reactive school closures were a major problem [22]. However, other studies have demonstrated the unintended consequences of school closure events, including difficulty arranging childcare, missed pay, and lower student performance on exams [5], [23]–[27]. Additionally, decisions to close schools can be difficult to communicate to parents, schools, and stakeholders [28]. While school closures are likely to be recommended in severe influenza pandemics and possibly in certain less severe pandemic scenarios to slow disease transmission in communities [4], for pre-pandemic preparedness it is important to consider the previous experience in communities with unplanned school closures and the ability of communities to cope with closure recommendations, as this may affect adherence to school closure and social distancing recommendations.

This study has certain limitations. The school closures in this study were limited to those reported through online media outlets or internet sites. It is likely that closure events for smaller schools or rural and remote areas are less represented in this study than larger urban schools. Because data collection depended on internet reports, there may be some misclassification of reasons for school closure as well as for dates of reopening. In this analysis, closure events where the durations of closure or the re-opening dates were not specified were assumed to be one-day closures; it is possible that this led to underestimation of the number of unplanned closure days for some school closure events. Finally, we used NCES data from 2010–2011 and 2011–2012 to obtain school and district characteristics; however, closure events reported were for the 2011–2012 and 2012–2013 school years. Thus, these databases may not have contained records for some newer or small schools, and they did not contain the most updated information for the 2012–2013 USCs.

To our knowledge, this is the first study describing the causes, frequency, and characteristics of all-cause USCs in the United States, and it highlights the impact of these events on students and communities. This study shows that USCs occur frequently but are typically of short duration. Most communities lack experience coping with longer USCs, with the exception of a few communities affected by relatively rare prolonged weather events or natural disasters. There was a large difference in the number of publicly announced USCs between the two years of this study; monitoring USCs over additional school years will help characterize this variation in USCs during non-pandemic years. The Community Preventive Services Task Force recommends preemptive and coordinated school closures in the event of a severe influenza pandemic to reduce or delay local spread of infection [4]. Public approval of school closure as a pandemic mitigation strategy was high during the 2009 H1N1 pandemic [29]; for a more severe pandemic, it is likely that communities will continue to find school closure policies acceptable, despite not having experience coping with prolonged closures. Although the consequences of any future prolonged preemptive school closures are unclear and community experience with USCs may change over time, a better understanding of the baseline scenario of school closures in the United States allows communities, educators, and public health officials to make more informed decisions in current preparations for school-related effects of major public health events, including a future pandemic.
